# Retrospective Analysis of Ultrasound-Guided Infraclavicular Block: Effect of Experience of Anesthesiologists on Volume of Local Anesthetic Administered

**DOI:** 10.1155/2019/4846956

**Published:** 2019-05-06

**Authors:** Tugce Yeniocak, Nur Canbolat

**Affiliations:** Department of Anesthesiology, Baltalimani Metin Sabanci Bone and Joint Diseases Education and Research Hospital, İstanbul 34470, Turkey

## Abstract

Performing a block under ultrasound guidance effectively requires skill; however, inexperienced anesthesiologists often use high-dose LA to ensure success. We aimed to share our experience with the ultrasound-guided infraclavicular brachial plexus block (USGICB) for upper extremity surgeries and to determine changes in failure rate and local anesthetic dose administered with gaining adequate experience. With approval from the local ethics committee, a retrospective review of records of 2953 patients who underwent USGICB between November 2011 and March 2015 was performed for evaluating the following data: age, sex, height, weight, operation type, American Society of Anesthesiologists physical status score, local anesthetic volume, complications, and success of USGICB. The patients were divided into 4 groups of 10 months each from November 2011 to March 2015: first 10-month period, 628 cases (group I); second 10-month period, 672 (group II); third 10-month period, 720 (group III); and the fourth 10-month period, 933 cases (group IV). Nine anesthesiologists with the same baseline experience in USG performed the blocks. During the initial period, when anesthesiologists had insufficient experience, local anesthetic (LA) dose, success rate, failed blocks, and complications were investigated. The LA volume administered in group I (33.7 ± 4.2 ml) was significantly higher than that in groups II, III, and IV (*p* < 0.05). Although a reduction in LA volume administered with increasing anesthesiologist experience was not statistically significant, a volume reduction of over 30 ml was observed in groups II, III, and IV compared with group I. Furthermore, in group I, failure rate (3.2%) was higher than that in groups II, III, and IV (*p* < 0.05). We concluded that sonographic guidance ensures a high success rate and that increased experience of anesthesiologists is associated with reduced complications and failure rate of blocks, in addition to prevention of LA overdose.

## 1. Introduction

Peripheral nerve blocks are widely used for surgical anesthesia and postoperative pain management for the elbow, forearm, wrist, and hand surgery [1]. Infraclavicular brachial plexus block (ICB) in upper extremity surgery has the advantages of high success rate and low risk of complications and ensures adequate analgesia, in addition to preventing tourniquet pain during surgery, ensuring patient comfort by allowing the patient to stay in a neutral position compared with the axillary block, preventing side effects of general anesthesia, requiring less needle insertions, being suitable for catheter insertion, contributing to postoperative analgesia, and shortening the duration to discharge [2–4]. In recent years, the use of ultrasound in the application of peripheral nerve blocks has facilitated nerve localization, detection of the boundaries of vascular structures and pleura, visual control of the needle tip, and monitoring of distribution of the injected volume of a local anesthetic (LA) [1]. Furthermore, the success rate of the block increased, and the complication risk, block performance time, number of needle insertions, and LA volume were reduced [5]. Performing a block under ultrasound guidance effectively requires hand-eye coordination, adequate knowledge of nerve anatomy, knowledge on USG physics, and skill.

Inexperienced anesthesiologists often use high-dose LA to ensure success. For ensuring that the appropriate dose of LA is administered, suitable technical training of the anesthesiologists is necessary. It has been reported that administration of a high dose of local anesthetic for peripheral nerve blocks by inexperienced anesthesiologists is associated with difficulties in identifying anatomic structures, ensuring sufficient local anesthetic distribution, and localization of the needle tip. The localization of the needle tip is usually facilitated by injecting minimal (0.5–1.0 ml) local anesthetic into the tissues [6]. Although reduction in the administered LA volume with increasing anesthesiologist experience has been reported for peripheral nerve blocks, the average number of attempts for ensuring consistency is unknown. Our primary goal was to share our experience with USGICB over 40 months and to determine changes in failure rate and LA dose administered with adequate experience.

## 2. Materials and Methods

After obtaining approval from the ethics committee of Baltalimani Bone Diseases Research and Training Hospital (Istanbul/Turkey), a retrospective review of anesthesia records on USGICB, from November 2011 until March 2015, was performed. Data on age, sex, height, weight, operation name, ASA score, LA dose, complications, and success rates were recorded. Inclusion criteria were ASA physical status classification I–IV, age 15 years or older, weight 50 kg or greater, and elective or acute elbow, forearm, wrist, or hand surgery. Patients with age <15 years or weight <50 kg and those who received bilateral blocks were excluded from the study.

After attending an ultrasound-guided regional anesthesia workshop, 9 anesthesiologists with the same baseline experience performed the USGICB. The blocks performed by inexperienced new anesthesiologists who participated on March 2015 were excluded, so the study was limited to 40 months. The patients were divided into 4 groups based on the number of months (10 months each) from November 2011 to March 2015.

Patients were prepared in a block room. Vascular access was established from the antecubital region at the opposite site of surgery; the patient was monitored using a 3-lead electrocardiogram (ECG), pulse oximetry, and noninvasive blood pressure monitoring. Hydration was maintained with an isotonic solution. After antiseptic preparation, all blocks were performed using a 15 MHz linear ultrasound probe (MyLabFive ESAOTE, Maastricht, Netherlands) and 100 mm 21G Stimuplex needle (B. Braun, Melsungen AG, Germany). The transducer was positioned below the clavicle in the deltopectoral area. Lateral, medial, and posterior cords of the brachial plexus were located around the axillary artery deep to the pectoralis minor muscle. The needle was inserted in-plane to the cephalic to the probe. The LA was injected into the lateral, posterior, and medial cords around the axillary artery. In all blocks, 0.5% bupivacaine +2% lidocaine mixture was administered as the LA solution (20–40 ml) with gentle aspiration after every 5 ml injection. After injection, when sufficient analgesia and motor block for the surgical procedure were obtained, patients were transferred to the operation room. Following routine monitoring, a pneumatic tourniquet was employed, and the surgical procedure began. In cases of inadequate analgesia, intravenous analgesic agent or general anesthesia was administered. Block failure was defined as sensation of pain, requirement for intravenous analgesics, or administration of general anesthesia during surgery. Pneumothorax, vascular puncture, LA toxicity, respiratory distress, and Horner syndrome were recorded as complications.

### 2.1. Statistical Analysis

Mean, standard deviation (SD), lowest median, frequency, and ratio values were used in descriptive statistics of the data. The distribution of variables was measured with the Kolmogorov–Smirnov test. Kruskal–Wallis and Mann–Whitney *U* tests were used to analyze the quantitative data. The qualitative data were analyzed using the chi-square test. SPSS 22.0 (IBM Corporation, Armonk, NY, USA) software was used to perform the statistical analysis.

## 3. Results

Between November 2011 and March 2015, data of 2953 patients that received USGICB were reviewed. In the 40-month period, patients were divided into 4 groups, each group comprising patients identified over 10 months: November 2011 to August 2012, 628 (Group I); September 2012 to July 2013, 672 (Group II); July 2013 to May 2014, 720 (Group III); and June 2014 to March 2015, 933 patients (Group IV). Demographic characteristics of the patients are presented in Table 1.

**Figure 1:**
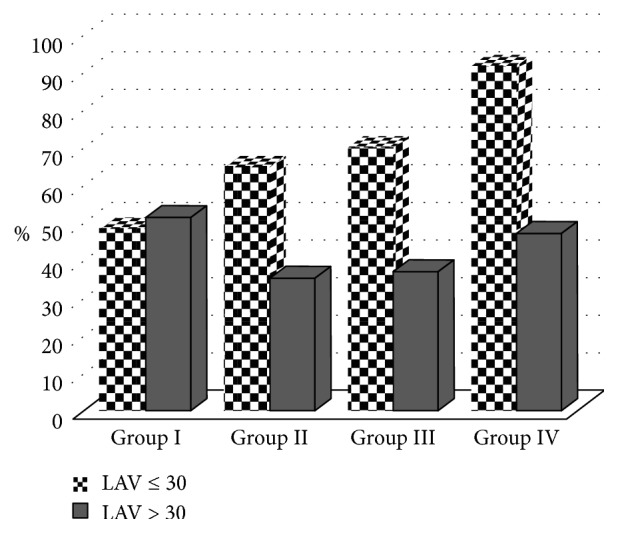
Comparison of local anesthetic volume administered in different study groups (≤30 ml and >30 ml).

**Figure 2:**
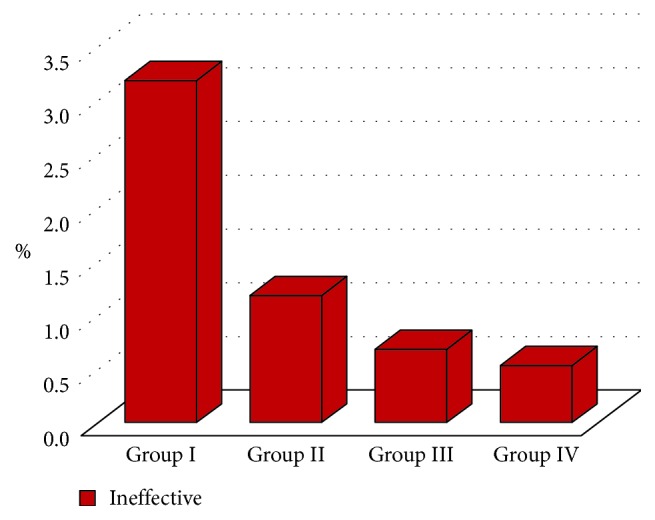
Comparison of the number of ineffective blocks in the study groups.

The LA volume administered in group I (33.7 ± 4.2 ml) was significantly higher than that in groups II, III, and IV (*p* < 0.05). The administered LA volume was similar in groups II, III, and IV (*p* > 0.05) (Table 2) (Figure 1). In group I, the number of patients administered an LA volume >30 ml was significantly higher than that in groups II, III, and IV (*p* < 0.05). In group II, the number of patients administered an LA volume >30 ml was significantly higher than that in group IV (*p* < 0.05). In groups II and III, the number of patients administered an LA volume >30 ml was similar (*p* > 0.05) (Table 2) (Figure 1). The failure rate was 3.2% (20) in group I, 1.2% (8) in group II, 0.7% (5) in group III, and 0.5% (5) in group IV. The failure rate in group I was significantly higher than that in groups II, III, and IV (*p* < 0.05). In groups II, III, and IV, the failure rate did not differ significantly (*p* > 0.05) (Table 2) (Figure 2). Complications such as pneumothorax, respiratory distress, syncope, or Horner syndrome were not detected in any patient.

## 4. Discussion

In this study, data on USGICBs performed in a 40-month period were reviewed retrospectively. As expected, the study findings revealed that the success rate of the block increased as the experience of the anesthesiologist increased. A decrease in LA volume administered was also detected in the same period. In our study, high failure rates and LA overdose were observed in the first 10-month period, which was considered as the novice period. In this study, 628 blocks were performed by 9 anesthesiologists, so each anesthesiologist performed an average of 69.7 ± 1.9 blocks during this period.

Ultrasound guidance is based on direct visualization of nerves, anatomic structures, and movement of the needle during insertion. Real-time monitoring of the needle insertion and local anesthetic distribution ensures the safety of the procedure. It helps to avoid complications and reduce the volume of LA administered [7]. The learning period of anesthesiologists for USG may be challenging. The success of a USG peripheral nerve block is operator dependent and demands cognitive and motor skill and adequate training and experience [8]. The exact number of attempts required to administer a successful USGICB is not known.

Bush and Mosteller established a mathematical learning model to estimate the average number of cases required to achieve a 95% success rate [9]. In many studies, these learning curves and mathematical models were used for basic skill development for using ultrasound, and the average number of procedures needed for acceptable success rates were determined [10–13]. In a study to determine the number of USG examinations required for measuring the fetal nasal bone, it was reported that 40 to 120 examinations are needed in the sonographer training period [11].

In our study, the success rate of USGICB increased from 96.8% to 99.5% in a 40-month period. De Oliveria Filho et al. found that 37 to 109 attempts were required to achieve a 95% success rate of USG regional anesthesia in a bovine phantom model [12]. In another study, with novice anesthesiologists performing a brachial plexus block, it was demonstrated that 70 blocks were required to obtain a 90% success rate [13]. Sites et al. reported that 80 blocks are needed to gain competency for visualizing the needle tip in peripheral blocks [14]. The number of attempts required for proficiency (low failure rate) according to our findings is similar to that reported in these studies [12–14].

Generally, the LA volume used for ICB is 20–40 ml [15]. In order to prevent LA toxicity, the LA volume was reduced from 80 ml (in 1980s) to 1 ml per nerve block under ultrasound guidance [16]. It is still unclear whether the LA concentration or the total LA dose is the primary determinant of success of the continuous peripheral nerve block. In our study, 20–40 ml LA (commonly 30 ml) was administered to the patients. The most preferred LA solutions for peripheral nerve blocks are 1.5–2% lidocaine, 2% mepivacaine, 0.5% bupivacaine, 0.5% levobupivacaine, and 0.5% ropivacaine [17]. In our study, as in the study by Gurkan et al. [18], a 0.5% bupivacaine and 2% lidocaine mixture was used in all blocks. Although the LA volume used in the novice period was up to 40 ml, in the second 10-month period, lower volume of LA was used with increase in experience.

Our study differs from other studies in that it contains a fairly large group of patients (2953 patients) that were administered USGICB. In the literature, no study on USGICB in a single center with such a large number of patients has been reported. Furthermore, our study differs from others in that the same technique and type of LA was used in each case, with the same level of knowledge and experience in USG, which provides information on the learning curve of anesthesiologists for USGICB.

This study has some limitations. First, the study started just after a USG course that the 9 anesthesiologists had attended. Therefore, it was assumed that they had the same baseline experience; however, personal abilities and experiences could not be assessed. The point at which the anesthesiologist is considered to be competent at performing this block was not clarified. Therefore, the average number of blocks that should be performed for attaining competency is unknown. More accurate results may be obtained by including more participants in future studies on this topic. Furthermore, new amateur colleagues joined the anesthesiologist team at March 2015, and we ended period to prevent the performance bias. Second, because of lack of information on the retrospective results, time to sensory block, block performance time, number of attempts for needle insertion, visual analog scale score, time to requirement for analgesic postoperatively, motor block duration, and errors during performance could not be evaluated. Third, due to the retrospective design of the study, common errors made by the anesthesiologists could not be recorded. In order to determine the average number of attempts for proficiency in learning curve studies, it is necessary to determine a cutoff value according to the success rate. Prospective studies including these parameters and evaluating each anesthesiologist's experience separately will produce more accurate results and allow the creation of learning curves.

## 5. Conclusions

We conclude that sonographic guidance ensures a high success rate of USGICB. Increased experience of anesthesiologists is associated with reduced complications and block failure, in addition to prevention of LA overdose. In further studies on USGICB performance and low-dose LA, it should be considered that, during the training period of anesthesiologists, outcomes of blocks may be influenced by individual skills. Performance studies of peripheral nerve blocks must be individualized for every technique and for different block areas, considering anatomic differences and performance difficulties. Considering this aspect, standardization of the techniques for peripheral blocks is essential to ensure proficiency.

## Figures and Tables

**Table 1:** Patient characteristics.

	Group I			Group II			Group III			Group IV			*p*
	Mean ± SD, *n* (%)		Median	Mean ± SD, *n* (%)		Median	Mean ± SD, *n* (%)		Median	Mean ± SD, *n* (%)		Median	
Age	38.2 ± 14.7		35	38.6 ± 14.9		37	35.9 ± 14.7		33	35.7 ± 14.1		33	**0.000**
*Sex*													
Female	227	36.1%		239	35.6%		215	29.9%		243	26.0%		**0.000**
Male	401	63.9%		433	64.4%		505	70.1%		690	74.0%		
BMI	26.3 ± 4.8		26	26.1 ± 4.9		26	26.2 ± 5.1		25	25.9 ± 4.6		25	0.417
*ASA*													
I	341	54.3%		331	49.3%		420	58.3%		633	67.8%		**0.041**
II	263	41.9%		324	48.2%		285	39.6%		285	30.5%		
III	22	3.5%		17	2.5%		14	1.9%		14	1.5%		
IV	2	0.3%		0	0.0%		1	0.1%		1	0.1%		

Data were analyzed using the Kruskal–Wallis (Mann–Whitney *U* test) or chi-square test.

**Table 2:** Comparison of local anesthetic volume (LAV) used and effectiveness of the ultrasound-guided infraclavicular brachial plexus block between groups I, II, III, and IV.

	Group I			Group II			Group III			Group IV			*p*
	Mean ± SD, *n* (%)		Median	Mean ± SD, *n* (%)		Median	Mean ± SD, *n* (%)		Median	Mean ± SD, *n* (%)		Median	
Local anesthetic volume (ml)	33.7 ± 4.2		35	32.7 ± 4.1		30	32.4 ± 3.8		30	32.0 ± 4.0		30	**0.000**
*LAV*													
≤30	305	48.6%		435	64.7%		471	70.1%		616	91.7%		**0.000**
>30	323	51.4%		237	35.3%		249	29.9%		317	8.3%		
*Result*													
Effective	608	96.8%		664	98.8%		715	99.3%		928	99.5%		**0.000**
Ineffective	20	3.2%		8	1.2%		5	0.7%		5	0.5%		

Data were analyzed using the Kruskal–Wallis (Mann–Whitney *U* test) or chi-square test.

## Data Availability

The data used to support the findings of this study are available from the corresponding author upon request.
